# Short and long-term effects of kidney donation on mineral and bone metabolism

**DOI:** 10.1186/s12882-024-03827-0

**Published:** 2024-10-26

**Authors:** Eduardo Jorge Duque, Gustavo Fernandes Ferreira, Ivone Braga Oliveira, Wagner Dominguez, Fabiana Agena, Vanda Jorgetti, Francine Lemos, Myles Wolf, Elias David-Neto, Rosa Maria A. Moysés

**Affiliations:** 1 Urology Division, Kidney Transplant Service – Hospital das Clínicas da Faculdade de Medicina da Universidade de São Paulo (HCFMUSP), São Paulo, Brazil; 2 Nephrology Division, Laboratório de Fisiopatologia Renal (LIM16) - Hospital das Clínicas da Faculdade de Medicina da Universidade de São Paulo (HCFMUSP), São Paulo, Brazil; 3 Nephrology Division, Department of Medicine, Duke Clinical Research Institute, Duke University School of Medicine, Durham, NC USA

**Keywords:** Kidney donation, Bone metabolism, Parathyroid hormone, FGF23, Calcium

## Abstract

**Background:**

Living kidney donors (LKD) experience an abrupt decline in glomerular filtration rate (GFR) resulting in abnormalities of mineral and bone metabolism (MBD), and this may have implications for skeletal health. We prospectively studied acute and long term MBD adaptation of LKD from two kidney transplant centers (São Paulo, Brazil and Miami, USA).

**Methods:**

Renal function and MBD parameters longitudinally after kidney donation (baseline – D0, day 1, 14, 180 and 360 post-operatively) were measured in 74 patients (40 y, 73% female, 54% Brazilian). A subset of 20 donors from Brazil were reassessed after 10 years of nephrectomy.

**Results:**

At baseline, Brazilian donors presented lower intact FGF23 (20.8 vs. 80.1 pg/mL, *P* < 0.01) and higher PTH (47.4 vs. 40.1, *P* = 0.04) than their US counterparts. GFR decreased to 63% of its baseline levels just after donation but improved 10% during the first year. PTH levels increased on D1, returning to baseline levels on D14, while FGF23 remained higher than baseline over the first year. LKD had a significant reduction of serum phosphate on D1, which returned to baseline levels on D180. A higher fractional excretion of phosphate (FEP) was noted since D14. After 10 years of donation, 20 LKD presented a sustained reduction in GFR (74.8 ± 14mL/min). There was a return to baseline in serum FGF23 [21.8 (18–30) pg/mL] and FEP, accompanied by an increase in serum calcium. PTH remained elevated (57.9 ± 18 pg/mL), whereas serum calcitriol and Klotho were lower than before the donation.

**Conclusions:**

The abrupt decline in kidney mass is associated with an increase in PTH and FGF23 that is not explained by phosphate retention. In a long-term evaluation, LKD showed a sustained drop in GFR, with lower serum calcitriol and Klotho, and higher PTH. The effects of these changes should be investigated in further studies.

**Supplementary Information:**

The online version contains supplementary material available at 10.1186/s12882-024-03827-0.

## Introduction

Living kidney donation is an important source of organs for patients with end-stage chronic kidney disease (CKD) worldwide. According to the Brazilian Registry of Transplants, living kidney donation corresponds to 14% of all kidney transplants in the last five years in Brazil. Although nephrectomy procedure is safe and contributes to better clinical outcomes for recipients, the living kidney donors (LKD) experience an abrupt decline in glomerular filtration rate and long-term evaluation of this population is scarce.

Disorders of mineral and metabolism (MBD) are frequent in patients with CKD, constituting an important cause of mortality and morbidity [1]. CKD leads to a consequent increase in parathyroid hormone (PTH) and fibroblast growth factor-23 (FGF23), mainly produced by osteoblasts and osteocytes, which inhibits phosphate tubular reabsorption, reduces 1α-hydroxylase activity and calcitriol levels [2]. The loss of renal function itself leads to a decrease in calcitriol production, inhibiting intestinal absorption of calcium and phosphate. All these adaptive mechanisms aim to maintain calcium and phosphate homeostasis.

The impact of nephrectomy on the physiology of adaptive mechanisms is not fully understood and continued surveillance of donation-attributable risks is required [3]. Clinical studies for evaluation of the consequences of kidney donation on CKD-MBD have several limitations, as there are few prospective studies with long-term follow-up and most reflect the results of a single center. This study aims to prospectively evaluate acute and long-term MBD adaptation in LKD of two different centers.

## Methods

This is a longitudinal, prospective study of patients ≥ 18 years of age who underwent nephrectomy for organ donation at the Hospital das Clínicas da Faculdade de Medicina da Universidade de São Paulo (FMUSP), Brazil and at the Jackson Memorial Hospital, Miami Florida, USA, between January 2010 and June 2011. Non-eligibility criteria for kidney donation included diabetes; body mass index (BMI) ≥ 35 Kg/m^2^; systemic hypertension (BP ≥ 140/80 mmHg or who were on medication for adequate control); estimated glomerular filtration rate (eGFR) less than 60 mL/min; microalbuminuria greater than 30 mg/day; or use of calcium supplements or vitamin D analogs.

**Fig. 1:**
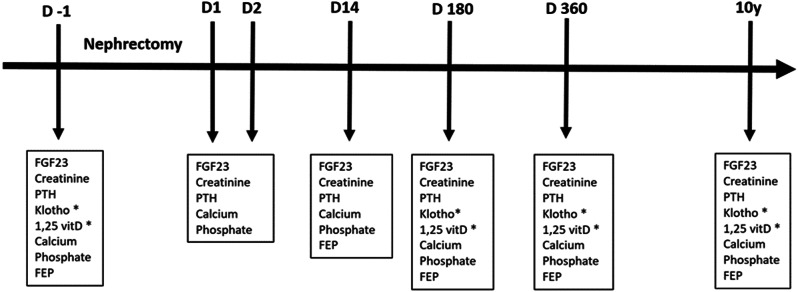
Study flowchart. FGF23, fibroblast growth factor 23; PTH, parathyroid hormone; 1,25 vitD, 1,25-dihydroxyvitamin D; FEP, fractional excretion of phosphate

Kidney donation for transplantation followed a standard local protocol, and transplant day was considered as D0. Fasting blood samples were collected from participants the day before (D-1) and prospectively after nephrectomy (D1, D2, D14, D180, D360, 10 years [20 cases]), as shown in Fig. 1. The samples were frozen and stored at − 80 °C. Urine was collected on the same days, except D1 and D2 for fractional excretion of phosphate (FEP) evaluation, which was calculated as 100 X ([urine phosphate/serum phosphate x serum creatinine/urine]). Creatinine, calcium and phosphate were measured by automated colorimetric method. PTH was measured by chemiluminescent immunoassay. Diasorin assay was used to measure 1,25-dihydroxyvitamin D (1,25(OH)_2_D). ELISA assays were performed to measure intact FGF23 (Immutopics) and Klotho (MicroVue, Quidel). The eGFR was calculated using the CKD-EPI (Chronic Kidney Disease Epidemiology Collaboration) 2021 equation. The institutional review boards at the participating sites, including Hospital das Clínicas (#0267/10) and Jackson Memorial Hospital, approved this study. All participants gave written informed consent.

Continuous variables were presented as mean and standard deviation or median and interquartile range, appropriately. Continuous variables were compared with a paired t-test or the Wilcoxon test for those with Gaussian and non-Gaussian distribution, respectively. Categorical variables are expressed as absolute values and percentages. Comparisons between populations were made using Mann-Whitney test and comparisons between frequencies were made using the chi-square test. Correlations between independent variables were quantified by calculating Pearson’s or Spearman’s coefficient, as appropriate. Values of *P* < 0.05 were considered statistically significant. Analyses were performed with the IBM SPSS Statistics software package, version 21.0 (IBM Corporation, Armonk, NY, USA) and GraphPad Prism software, version 9.0 (GraphPad Software, Inc., San Diego, CA, USA).

## Results

Between January 2010 and June 2011, 78 patients who met the inclusion criteria were recruited, 3 did not undergo nephrectomy due to clinical issues and 1 withdrew consent after the surgery. The results of 74 patients (up to D14), 70 patients (D180) and 64 patients (D360) were analyzed. Baseline characteristics are shown in Table 1. A subset of 20 donors from Brazil, who remained monitored by the transplant team, were reevaluated after 10 years of nephrectomy.

**Table 1:** Baseline demographic, clinical, surgical and laboratory data

Baseline Characteristics	Overall	Brazil	US	*P*
Age, years	40 ± 11	41 ± 9	40 ± 13	0.69
Female, %	73%	80%	64.7%	0.67
White, %	76%	77.5%	73.5%	0.08
Body weight, Kg	72.3 ± 12	67.8 ± 9	77.5 ± 14	**0.0006**
BMI, Kg/m^2^	26.4 ± 3.6	25.9 ± 3.2	26.9 ± 4	0.24
**Surgery**				
Laparoscopic, %	68.9%	79.4%	60%	0.24
Left kidney, %	58.1%	79.4%	40%	0.55
**Kidney function**				
eGFR, mL/min/1.73 m^2^	109.5 ± 21.3	101.2 ± 13	101 ± 15	0.97
Creatinine, mg/dL	0.81 ± 0.1	0.79 ± 0.1	0.83 ± 0.2	0.21
**Mineral Metabolism**				
Calcium, mg/dL	9.4 ± 0.5	9.1 ± 0.7	9.5 ± 0.4	**0.01**
Phosphate, mg/dL	3.7 ± 0.5	3.6 ± 0.5	3.8 ± 0.4	0.06
PTH, pg/ml	44.4 ± 14.7	47.4 ± 15	40.7 ± 12	**0.04**
Fractional excretion of phosphate,%	11.4 ± 5.2	11 ± 3.9	11.5 ± 6.5	0.91
FGF-23, pg/ml	44.4 (19–80)	20.8 (14–30)	80.1 (64–95)	**0.0002**

BMI, Body Mass Index; eGFR, Estimated Glomerular Filtration Rate; PTH, parathyroid hormone; FGF23, fibroblast growth factor 23. * All Brazilians were considered Hispanic

Reference values: BMI, 18–25 Kg/m^2^; Calcium, 8.6–10.2 mg/dL; Phosphate, 2.7–4.5 mg/dL; Parathormone, 16–87 pg/mL; FEP, 5–18%; FGF-23, 19–81 (percentile 25–75) Data are presented as mean ± SD, *n* (%), or median (IQR). P Brazilian vs. American patients

**Fig. 2:**
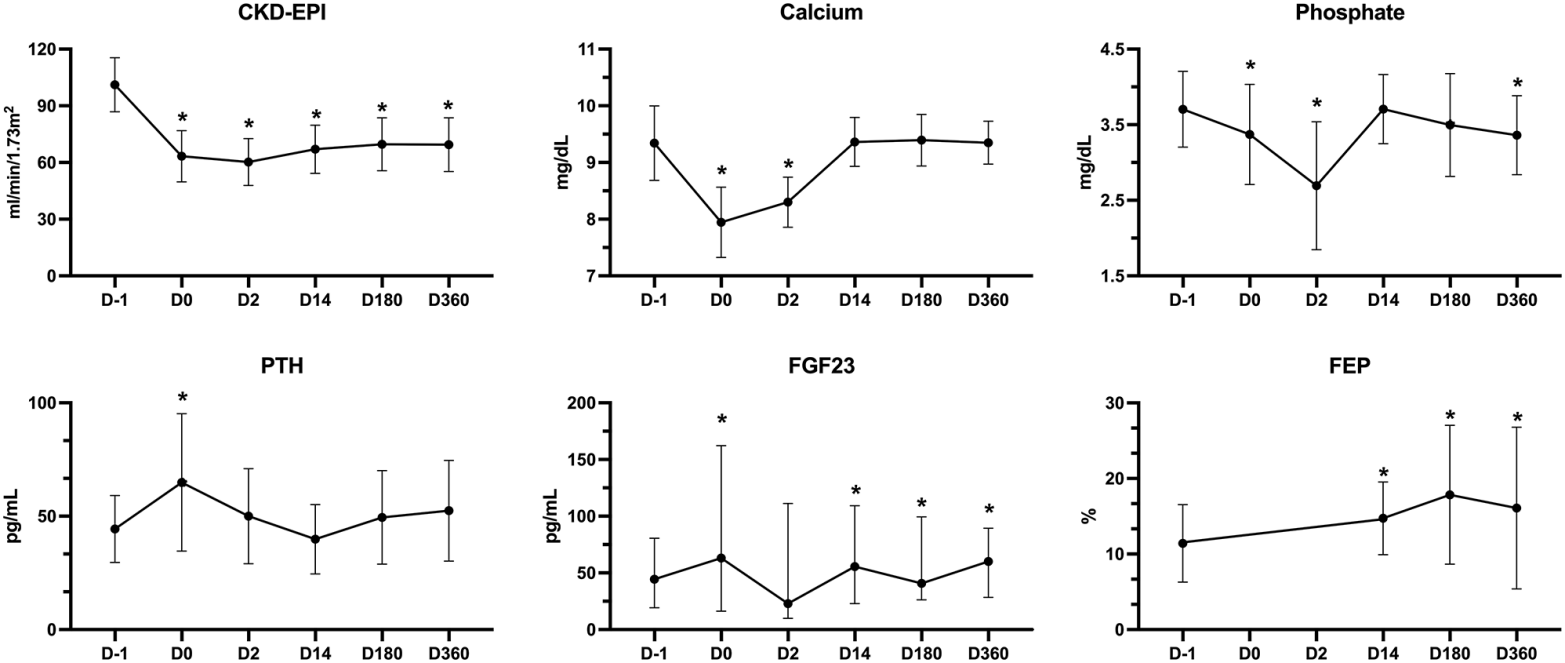
Variation in Glomerular Filtration Rate (**A**), Calcium (**B**), Phosphate (**C**), PTH (**D**), FGF23 (**E**), and FEP (**F**) over the first year after kidney donation. PTH, parathyroid hormone; FGF23, fibroblast growth factor 23; FEP, fractional excretion of phosphate. Data are presented as mean ± SD or median (IQR). * *P* < 0.05

Before nephrectomy, all parameters were within reference limits, except 3 patients (4%) who presented eGFR between 60 and 80 ml/min/1.73m^2^. Figure 2; Table 2 show the evolution of laboratory parameters during the first year after donation. eGFR reduced 37% on D2 and started recovery from D14 with no difference on D180 and D360. eGFR after one year of kidney donation represented 69% of the initial value and, despite significantly lower than baseline, improved around 10% compared to D2. Six patients (9.4%) had GFR less than 60 ml/min/1.73m^2^.

**Table 2:** Changes in renal function and CKD-MBD parameters of 74 kidney donors over the first year

	D -1	D 1	D 2	D 14	D 180	D 360
eGFR (mL/min/1,73 m2)	101.1 ± 14	63.3 ± 13***	60.2 ± 12***	67 ± 13***	69.9 ± 14***	69.4 ± 14***
Creatinine (mg/dL)	0.81 ± 0.14	1.24 ± 0.24***	1.27 ± 0.67***	1.18 ± 0.22***	1.14 ± 0.22***	1.13 ± 0.22***
Calcium (mg/dL)	9.4 ± 0.5	7.9 ± 0.5***	8.3 ± 0.4***	9.4 ± 0.4	9.4 ± 0.5	9.3 ± 0.4
Phosphate (mg/dL)	3.7 ± 0.5	3.3 ± 0.6***	2.6 ± 0.5***	3.7 ± 0.5	3.5 ± 0.6	3.3 ± 0.5***
PTH (pg/mL)	44.4 ± 14.7	64.9 ± 30.3***	50 ± 21	39.9 ± 15.3	49.5 ± 20.6	52.4 ± 22.2
FGF23 (pg/mL)	44.4 (19–80)	63.1 (16–162)*	22.8 (10–111)	55.6 (23–109)***	40.7 (26–99)	60.1 (28–89)***
FEP (%)	11.4 ± 5.2	NA	NA	14.7 ± 4*	17.8 ± 9.1***	15.2 ± 8.1**

eGFR, estimated Glomerular Filtration Rate; FGF23, fibroblast growth factor 23; PTH, parathyroid hormone; FEP, fractional excretion of phosphate

NA; unchecked *P < 0.05; **P < 0.01; ***P < 0.001 compared to D -1 Data are presented as mean ± SD, *n* (%), or median (IQR)

Immediately after nephrectomy, FGF23 and PTH increased accompanied by a drop in serum calcium and phosphate. After 14 days, calcium, phosphate and PTH levels returned to baseline values. Only 2 patients (3.1%) had PTH levels higher than the reference values at the end of first year.

Although within the reference range, after one year, serum phosphate levels were lower than at baseline. FEP measured from D14 showed an increase around 31% and remained higher than baseline on D360. In a similar way, FGF23 increased on the first day after nephrectomy and remained higher on D360. Delta FGF23 correlated positively with delta phosphate on D14 and D360 (*r* = 0.26; *P* < 0.05), and negatively with eGFR on D180 (*r* = 0.34; *P* < 0.01).

**Fig. 3:**
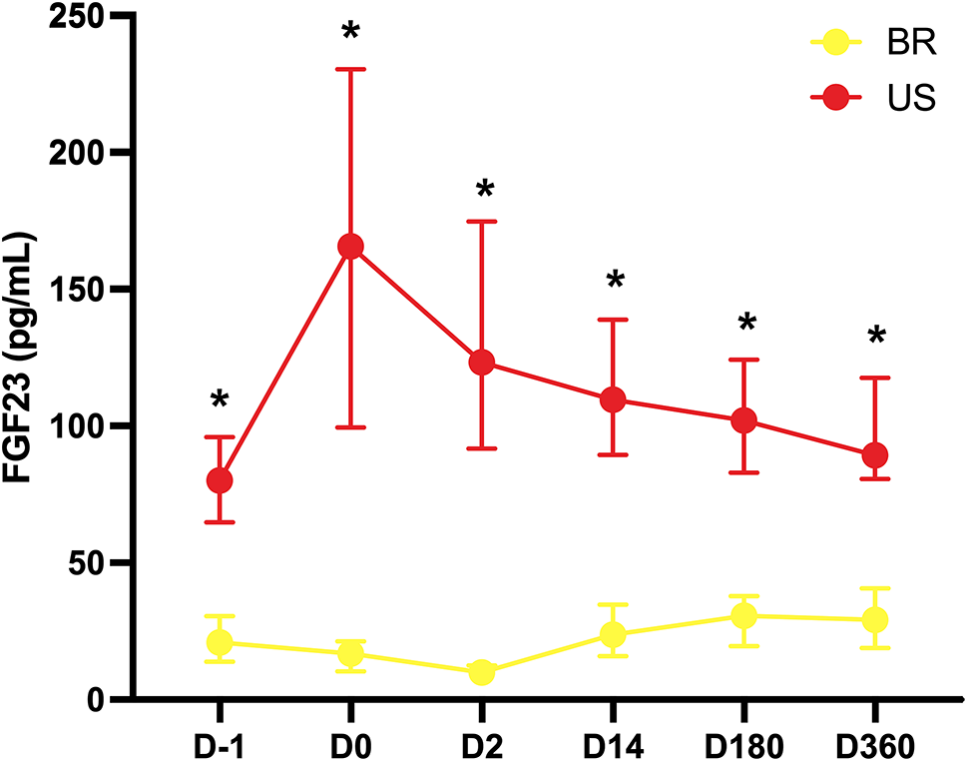
FGF 23 levels evolution over the first year after kidney donation by country. * *P* < 0.05 BRA vs. USA

There were differences between the two centers involved in this analysis (Supplementary Tables 1 and Fig. 3). At baseline, Brazilian donors presented lower FGF23 (20.8 vs. 80.1 pg/mL, *P* = 0.0002) and higher PTH (47.4 vs. 40.1 pg/mL, *P* = 0.04) than their American counterparts. Brazilian donors ended the first year with PTH and FEP above the baseline values. Otherwise, American donors presented lower calcium levels within the normal range on D360, and higher FGF23 (Fig. 3), with no difference in PTH and FEP. In both groups, phosphate was lower after one year of donation.

## Long-term evaluation

**Fig. 4:**
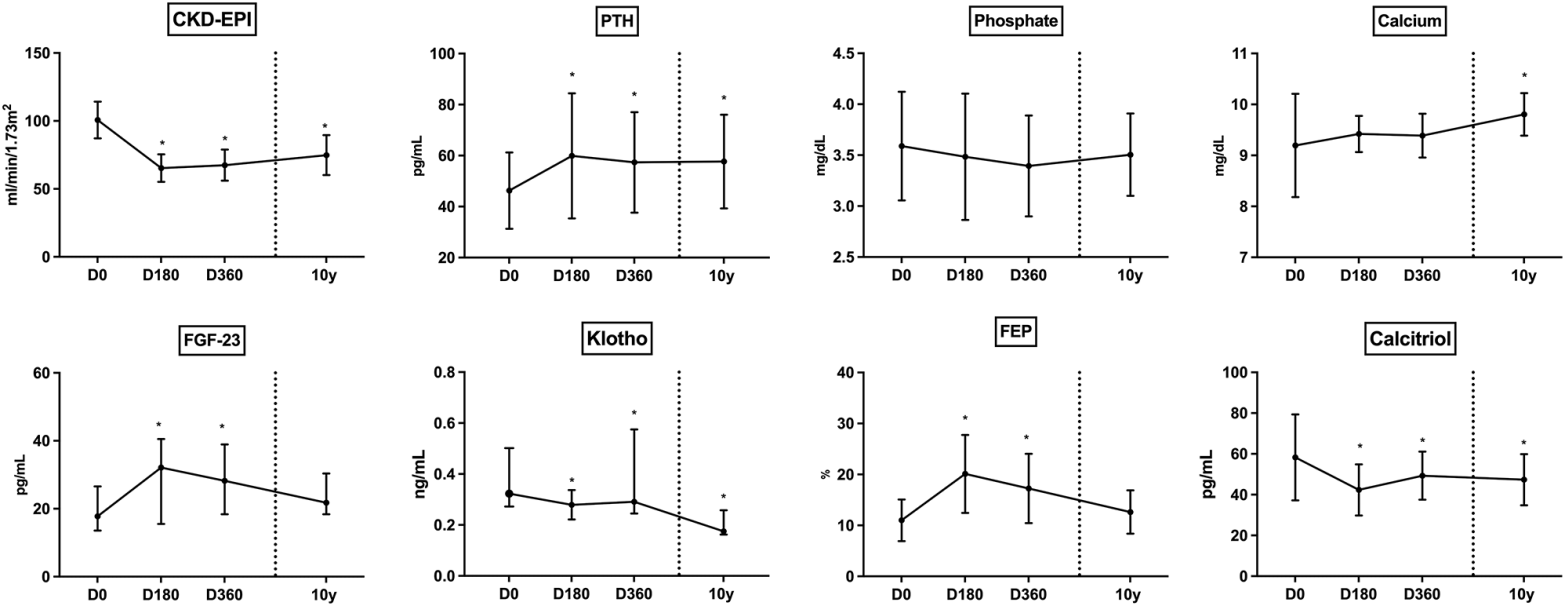
Changes in renal function and CKD-MBD parameters of 20 Brazilian donors over 10 years. PTH, parathyroid hormone; FGF23, fibroblast growth factor 23; FEP, fractional excretion of phosphate. Data are presented as mean ± SD or median (IQR). * *P* < 0.05

MBD analysis after 10 years of Brazilian LKD is shown in Table 3; Fig. 4. The mean age was 53 ± 10 years, and donors presented a sustained reduction in GFR, soluble Klotho, and calcitriol. In this subset of patients, PTH remained higher than baseline, although within the reference range, whereas serum calcium increased. FEP reduced along with FGF23, and phosphate levels remained stable.

**Table 3:** Changes in renal function and CKD-MBD parameters of 20 kidney donors in long term follow-up

	D -1	D 180	D 360	10 years
eGFR (ml/min/1,73m^2^)	100.6 ± 13	65.2 ± 10***	67.4 ± 11***	74.8 ± 14***
Calcium (mg/dL)	9.3 (8.9–9.8)	9.4 (9.2–9.7)	9.3 (9-9.7)	9.8 (9.5–10)*
Phosphate (mg/dL)	3.6 ± 0.5	3.5 ± 0.6	3.4 ± 0.5	3.5 ± 0.4
PTH (pg/ml)	46.2 ± 15	59.9 ± 24*	57.3 ± 19*	57.9 ± 18*
FGF23 (pg/mL)	17.7 (13.5–26.5)	32 (15.5–40.5) *	28.2 (18.3–39) *	21.8 (18.3–30.3)
1,25-vit D (pg/mL)	55.2 (39.3–62.7)	43.3 (34.7–51.1)***	48.8 (43.7–60.3)	46.9 (41.3–57.6)*
Klotho (ng/mL)	0.33 (0.27–0.56)	0.28 (0.22–0.43)*	0.29 (0.26–0.63)*	0.17 (0.16–0.31)*
FEP (%)	10 (9–13)	18.5 (15.7–21.7)***	16.5 (13.2–21.7)*	13.3 (10.4–15)

eGFR, estimated Glomerular Filtration Rate; FGF23, fibroblast growth factor 23; PTH, parathyroid hormone; FEP, fractional excretion of phosphate; *P < 0.05 *** *P* < 0.001 compared to D -1

Data are presented as mean ± SD, *n* (%), or median (IQR)

## Discussion

The adaptive response to the loss of 50% of kidney mass begins just after nephrectomy and, in general, the recovery starts a few months after surgery [4]. However, the abrupt decline in GFR after kidney donation also results in abnormalities of mineral and bone metabolism, and this may affect skeletal health. This prospective study evaluated acute changes of MBD parameters and, in a subgroup of patients, how they adapted in the long-term follow-up.

In the scenario of CKD-MBD, some evidence has reported variations in calcium and phosphate levels after kidney donation, as well as in mineral biomarkers, such as PTH and FGF23. In a case-control study with 101 donors, Seyahi et al. have shown that LKD had similar PTH, but lower calcium and phosphate levels after 5 years of nephrectomy compared to healthy volunteer controls [5]. Another case-control analysis with similar follow-up has revealed that 198 kidney donors, compared to 98 healthy volunteers, presented higher PTH, FGF23 and FEP, and lower levels of phosphate and calcitriol [6]. Our patients presented an acute reduction of calcium and phosphate levels and, at the end of the first year after donation, calcium returned to baseline levels and phosphate remained slightly reduced. The decrease in serum calcium and phosphate levels on the first day must be associated not only with the reduction of renal mass, but also with excessive hydration and fasting related to the surgical procedure. Hypocalcemia and higher PTH are well-described phenomena in abdominal surgeries and are not specific to unilateral nephrectomies [7]. All patients in this study developed a rapid decline in total calcium values, with their nadir on the first day after nephrectomy. Hypocalcemia is responsible for PTH stimulation, which leads to progressive recovery of serum calcium levels, but increases urinary phosphate loss. In addition to the preoperative steps, the behavior of FGF23 and PTH induced an increase in phosphaturia and consequent hypophosphatemia. However, it has been described that FGF23 also increases with inflammation, such as surgery-related acute kidney injury [8], and this could be associated with AKI progression [9]. In our patients, the surgery itself, besides the loss of renal mass could have also contributed to the FGF23 increase.

Kidney donors are discharged from hospitals around the third day after donation, probably returning to their usual eating habits as they are not asked to restrict their diets. We observed a significant drop in GFR and higher phosphaturia despite normal phosphate levels from D14 on. Then, we assume that phosphate intake remains the same as pre-donation but with a lower GFR, and during the first year, to keep phosphate levels stable, the increase of FGF23 and FEP are required.

In a previous publication, phosphate was shown to acutely reduce and then normalize 6 weeks after donation without acute changes in FGF23 and PTH [10]. Despite the phosphate reduction, FGF23 rose remarkably on D1 in our cohort. In fact, it has been already shown that not only phosphate, but inflammatory mediators could drive the bone derived FGF23 [11]. The behavior of FGF23 after donation remains controversial based on published data. In a previous prospective study with 9 patients, FGF23 increased after 1 week of kidney donation and returned to baseline values after 6 months [12]. A prospective observational study of 29 patients undergoing nephrectomy due to urological indications has not demonstrated variation in c-terminal FGF23 after surgery [13]. Another prospective evaluation of 27 donors showed a reduction in intact FGF23 levels in the first 3 consecutive days after nephrectomy [14]. Conversely, Kasiske et al. have evaluated 182 donors and showed that FGF23 is higher even 3 years after donation compared to controls [15]. It should be highlighted that the heterogeneity of the FGF23 assays in each study might influence these distinct findings.

FEP is considered a surrogate marker of mineral bone metabolism disturbance after donation, and its increase is influenced by pre-donation FEP and decline in eGFR [16]. We observed an increase in FGF23 levels from D14 to D360, and there was a positive correlation between FGF23 and FEP on D14 and 360. On D14, PTH levels returned to normal, but FGF23 remained higher over the first year, which could partially explain higher FEP up to D360.

MBD parameters had differences at baseline and during the follow-up according to the center where donors were located. Brazilian donors presented lower levels of FGF23 and higher levels of PTH compared to those from the US. Diet content could explain this, as an energy-dense diet, which induces a pro-inflammatory state, and dietary phosphate have been demonstrated to stimulate FGF23 production [17–19]. Moreover, environmental factors were also shown to influence PTH [20, 21], which could explain the distinct variation of PTH between the cohorts after one year of donation.

Investigation for long-term evaluation of CKD-MBD in kidney donors is scarce and has several limitations due to the heterogeneity of studies. Gossmann et al. have published a retrospective study with 135 patients, revealing an increase in PTH and a decrease in 1,25(OH)_2_D eleven years after donation [22]. A prospective controlled study has shown that PTH concentrations increased, phosphate reduced, and calcium levels were unchanged 3 years after donation [23]. Although a retrospective study did not find evidence of increased fragility fracture risk in LKD [24], higher concentration of resorption and bone formation markers were observed in donors compared to healthy controls [15, 25].

It has been already demonstrated that 1,25(OH)_2_D declines after donation, PTH increases, reducing phosphate levels, and Klotho levels are lower after one year of donation [14]. Indeed, Klotho was previously shown to be acutely reduced since the first day up to the end of the first year after donation [10]. α-Klotho is primarily synthesized in the kidneys and has an important role on mineral metabolism homeostasis [26, 27]. Serum klotho levels increase early after successful transplant and decrease after living donation, compared to baseline respectively [28], and the influence of pretransplant soluble klotho levels on post-kidney transplant soluble Klotho has been shown in both living donors and kidney recipients [29]. However, there is conflicting information about the changes in klotho after donation. Thorsen et al. did not find a difference in serum α-Klotho compared to healthy controls 5 years after donation [30]. In our patients, the long-term analysis revealed that soluble klotho and calcitriol reduced significantly after 10 years compared to baseline, following the reduction of the eGFR. Curiously, PTH stabilized at a higher-level despite being within reference values, and FGF23 dropped in the long-term evaluation, returning to baseline levels, as did the FEP. We wonder whether these changes occur as adaptive strategies to maintain skeletal homeostasis.

The results of this study should be cautiously interpreted. We are aware of several limitations that include the patient’s dropout due to lost to long follow-up which resulted in a relatively small sample size, the lack of bone remodeling markers, fracture risk evaluation or densitometry analysis. Moreover, some MBD parameters, such as 25OH vitamin D and 1,25(OH)_2_D, have not been assessed in all patients. Considering the challenge of monitoring LKD over a long period, our strength includes the fact this is the longest prospective evaluation focused on MBD evolution in a cohort of kidney donors.

In conclusion, nephrectomy in healthy patients led to changes in MBD parameters, initially increasing FGF23 levels and FEP, which decreased to baseline level in the long-term analysis. Despite the stability of phosphate levels, there was an increase in calcium and PTH levels, in addition to a reduction in soluble klotho and calcitriol levels, which may be associated with a reduction in eGFR.

## Electronic supplementary material

Below is the link to the electronic supplementary material.

Supplementary Material 1

## Data Availability

No datasets were generated or analysed during the current study.

## Abbreviations

LKD: Living kidney donors
GFR: Glomerular filtration rate
MBD: Mineral and bone metabolism
FEP: Fractional excretion of phosphate
CKD: Chronic kidney disease
PTH: Parathyroid hormone
FGF23: Fibroblast growth factor-23
1,25(OH)_2_D: 1,25-dihydroxyvitamin
BMI: Body mass index
