# Supplementary material for: Short and long-term effects of kidney donation on mineral and bone metabolism
Source: BMC Nephrol. 2024 Oct 26;25:381. doi: 10.1186/s12882-024-03827-0 (PMC11514742; doi:10.1186/s12882-024-03827-0)
Supplement: Supplementary file 1 — Supplementary Material 1 [file 12882_2024_3827_MOESM1_ESM.docx]

Supplementary Table 1. Changes in renal function and CKD-MBD parameters of kidney donors over the first year by country

|  |  | **D -1** |  | |  | **D 1** |  | **D 2** | |  | | **D 14** | |  | **D 180** | |  | | **D 360** | |  |
| --- | --- | --- | --- | --- | --- | --- | --- | --- | --- | --- | --- | --- | --- | --- | --- | --- | --- | --- | --- | --- | --- |
|  | **BRA** | **USA** | *P* | | **BRA** | **USA** | *P* | **BRA** | **USA** | *P* | | **BRA** | **USA** | *P* | **BRA** | **USA** | | *P* | **BRA** | **USA** | *P* |
| eGFR  (mL/min/1,73 m2) | 101.2 ± 13 | 101 ± 15 | | 0.97 | 60.4 ± 10*** | 66.6 ± 16*** | **0.04** | 59.3 ± 9*** | 61.6 ± 16 *** | | 0.45 | 68.2 ± 11 *** | 65.5 ± 14*** | 0.35 | 70.1 ± 13*** | 69 ± 15*** | | 0.74 | 68.8 ± 11*** | 70.1± 16*** | 0.69 |
| Creatinine (mg/dL) | 0.79 ± 0.1 | 0.83 ± 0.2 | | 0.21 | 1.25 ± 0.2*** | 1.24 ± 0.3*** | 0.83 | 1.27 ± 0.2*** | 1.45 ± 0.1*** | | 0.24 | 1.13 ± 0.2*** | 1.23 ± 0.2*** | **0.04** | 1.1 ± 0.2*** | 1.18 ± 0.2*** | | 0.11 | 1.1 ± 0.2*** | 1.15 ± 0.2*** | 0.48 |
| Calcium (mg/dL) | 9.17 ± 0.7 | 9.55 ± 0.4 | | **0.01** | 7.9 ± 0.6*** | 7.9 ± 0.5*** | 0.92 | 8.2 ± 0.4*** | 8.3 ± 0.5*** | | 0.72 | 9.3 ± 0.4 | 9.8 ± 0.8 | **0.03** | 9.3 ± 0.4 | 9.5 ± 0.4 | | 0.11 | 9.4 ± 0.4 | 9.2 ± 0.3** | 0.2 |
| Phosphate (mg/dL) | 3.6 ± 0.5 | 3.8 ± 0.5 | | 0.06 | 3.2 ± 0.5** | 3.5 ± 0.7* | 0.12 | 2.7 ± 1*** | 2.5 ± 0.5*** | | 0.25 | 3.6 ± 0.4 | 3.8 ± 0.5 | **0.01** | 3.3 ± 0.6* | 3.7 ± 0.7 | | **0.01** | 3.2 ± 0.4** | 3.4 ± 0.6** | 0.13 |
| Parathormone (pg/mL) | 47.4 ± 15 | 40.1 ± 12 | | **0.04** | 52 ± 20 | 79 ± 34*** | **<0.01** | 49 ± 21 | 53.6 ± 24** | | 0.47 | 43 ± 15 | 35 ± 13 | **0.01** | 55 ± 24 | 43 ± 15 | | **0.03** | 58 ± 24* | 45.5 ± 17 | **0.01** |
| FGF23 (pg/mL) | 20.8 (14-30) | 80.1 (65-95) | | **<0.01** | 16.8 (10-21) | 165 (100-225)*** | **<0.01** | 10 (9-12) | 123 (91-174)** | | **<0.01** | 23.6 (16-34)*** | 109 (89-137)** | **<0.01** | 30.6 (21-36) | 102 (83-124) | | 0.12 | 29 (19-40)*** | 89 (80-117) | **<0.01** |
| FEP (%) | 11.3 ± 4 | 11.5 ± 6 | | 0.91 | NA | NA |  | NA | NA | |  | 16.7 ± 4*** | 11.3 ± 3 | **<0.01** | 19.4 ± 8*** | 15.5 ± 9 | | 0.1 | 17.5 ± 6*** | 14.6 ± 13 | 0.29 |

eGFR, estimated Glomerular Filtration Rate; FGF23, fibroblast growth factor 23; FEP, fractional excretion of phosphate;

NA; unchecked *P<0.05; **p<0.01; ***p<0.001 compared to D -1; *P*: BRA vs USA. Data are presented as mean ± SD, *n* (%), or median (IQR).
